# Serum visfatin level in β-thalassemia and its correlation with disease severity

**DOI:** 10.25122/jml-2023-0354

**Published:** 2024-03

**Authors:** Himadri Shukla, Anurag Singh, Rashmi Kushwaha, Shailendra Prasad Verma, Nishant Verma, Uma Shankar Singh

**Affiliations:** 1 Department of Pathology, King George Medical University, Lucknow, India; 2 Department of Clinical Hematology, King George Medical University, Lucknow, India; 3 Department of Pediatrics, King George Medical University, Lucknow, India

**Keywords:** beta-thalassemia, hematological disorders, visfatin, inflammation

## Abstract

Thalassemia is a group of genetic hematological conditions characterized by the defective synthesis of one or more hemoglobin chains. This genetic anomaly alters globin chain balance, causing hemolysis, ineffective erythropoiesis, and chronic inflammatory diseases. The proinflammatory adipocytokine visfatin is predominantly produced in visceral adipose tissue. Its evaluation in individuals with thalassemia may provide valuable insights into the assessment of disease severity. The aim of this study was to investigate the potential role of visfatin in the development of β-thalassemia and its association with the severity of the illness. The study included 40 patients with β-thalassemia and ten healthy individuals matched by age and sex. Serum visfatin level was measured using ELISA. We found that individuals with β-thalassemia major had significantly higher levels of serum visfatin than those with β-thalassemia minor and the control group (*P* < 0.001). A receiver operating characteristic curve revealed that serum visfatin levels were different in the three groups. Our results suggest that the serum level of visfatin is significantly correlated with the severity of β-thalassemia.

## INTRODUCTION

β-thalassemias are a group of inherited hematological diseases characterized by the defective synthesis of β-globin chains in hemoglobin. This deficiency gives rise to a spectrum of phenotypes, which can range from no clinical symptoms to severe anemia [1]. Two primary types of β-thalassemia have been identified: β-thalassemia major (ThM) and β-thalassemia minor, also known as β-thalassemia trait (ThT) [2,3]. The defective synthesis of β-globin chains results in an overabundance of α-globin chains, which in turn leads to impaired red blood cell production, hemolysis, and inflammation [4]. Patients with β-thalassemia exhibit a persistent state of inflammation characterized by elevated levels of inflammatory cytokines, including leptin, resistin, and visfatin. The involvement of proinflammatory cytokines has an important role in the progression of complications in individuals with β-thalassemia [5–7]. Adipocytokines are widely recognised as significant contributors to the development and progression of various vascular and inflammatory conditions [8]. Visfatin, a recently identified adipocytokine with a molecular weight of 52 kDa, is produced by visceral adipose tissue and has been linked to inflammatory and vascular disorders. It can stimulate the production of proinflammatory cytokines, including interleukin (IL)-1, tumor necrosis factor α (TNF-α), and IL-6. As an insulin-mimicking adipocytokine, visfatin also affects several distinct processes, including immunity, insulin resistance, endothelial cell function, and angiogenesis [9,10]. There are only a handful of studies that have investigated the potential relationship between visfatin and β-thalassemia. Consequently, the aim of this study was to examine the role of visfatin in patients with different types of β-thalassemia and its association with the severity of the disease.

## MATERIAL AND METHODS

The present study was carried out at the Department of Pathology, Department of Clinical Haematology, and the Department of Paediatrics of King George’s Medical University in Lucknow, India, between January 2022 and December 2022. We included 40 patients diagnosed with thalassemia (12 patients with ThM and 28 patients with ThT) and a control group of ten healthy individuals matched by age and sex. The diagnosis of β-thalassemia was established with the help of complete blood count and peripheral blood smear examination, the analysis of data from high-performance liquid chromatography, as well as the assimilation of clinical findings. The study excluded patients who had hemoglobinopathies other than β-thalassemia.

We excluded patients with hemoglobinopathies other than β-thalassemia; other inflammatory diseases, such as rheumatoid disorders, myositis, panniculitis, and multisystem inflammatory syndrome; infectious, chronic, or genetic diseases; as well as those who were using drugs other than iron chelators.

The iron chelator deferasirox was administered to all patients diagnosed with β-thalassemia major at a dose of 15–35 mg/kg/day. Each patient underwent a thorough clinical evaluation, including a comprehensive medical history review, meticulous clinical assessment, complete blood count analysis, and examination of peripheral blood smears. Serum ferritin, soluble transferrin receptor (sTfR), and visfatin levels were assessed in all study participants using ELISA.

After centrifuging the blood for 15 min at 3,000 rpm, the serum was extracted, divided into aliquots, and kept at −70 °C until analysis. Red blood cell indices were assessed using a standard automated technique with an ADVIA 2120i system (Siemens). Serum ferritin levels were measured with a Cobas 6000 auto-analyzer (Roche-Hitachi), using the electrochemiluminescence method. Enzyme immunoassays for visfatin and sTfR were performed using ELISA kits CK-bio-13955 and CK-bio-13766, respectively, according to the manufacturer’s instructions.

### Statistical analysis

Statistical analysis was performed using SPSS 23 for Windows (IBM Corp). Continuous variables were expressed as mean ± s.d. or range. Means among groups were compared using analysis of variance (ANOVA). A *P* value of <0.05 was considered statistically significant.

## RESULTS

The present study comprised a total of 40 patients diagnosed with thalassemia and ten healthy controls. Patients with thalassemia were further divided into two subgroups: 12 patients with ThM and 28 patients with ThT. In the ThM group, 11 out of 12 patients (91.67%) were male, in the ThT group 14 out of 28 patients (50%) were male, and in the control group eight out of ten patients (80%) were male. The mean age of the participants was 1.30 ± 1.32 years in the ThM group, 28.50 ± 4.83 years in the ThT group, and 17.34 ± 7.32 years in the control group (*P* < 0.001) (Table 1).

**Table 1:** Distribution of study participants by age and sex

	Total (*n* = 50)	ThM group (*n* = 12)	ThT group (*n* = 28)	Control group (*n* = 10)
Female, *n* (%)	17	1 (8.33%)	14 (50%)	2 (20%)
Male, *n* (%)	33	11 (91.67%)	14 (50%)	8 (80%)
Male:female ratio	1.94:1	11.0:1	1:1	4.0:1

Hemoglobin levels were the highest in the control group (14.81 ± 1.65 g/dl), followed by the ThT (11.31 ± 1.61 g/dl) and the ThM group (5.17 ± 2.33 g/dl). A significant difference was noted between the different groups (*P* < 0.001). The mean number of red blood cells was the highest in the ThT group (5.39 ± 0.72 million cells per mm^3^), followed by the control group (4.58 ± 0.43 million cells per mm^3^), and the ThM group (2.28 ± 0.96 million cells per mm^3^). There was a significant difference between the different groups (*P* < 0.001) (Table 2).

**Table 2:** Comparison of mean hematological parameters

	ThM group (*n* = 12)	ThT group (*n* = 28)	Control group (*n* = 10)	*P* value
Hemoglobin (g/dl)	5.17 ± 2.33	11.31 ± 1.61	14.81 ± 1.65	<0.001
Red blood cells (× 10^6^/mm^3^)	2.28 ± 0.96	5.39 ± 0.72	4.58 ± 0.43	<0.001
Mean corpuscular volume (fl)	66.68 ± 14.14	65.07 ± 6.34	95.29 ± 5.06	<0.001
Mean corpuscular hemoglobin (pg)	22.61 ± 3.01	21.38 ± 1.89	31.80 ± 1.51	<0.001
Mean corpuscular hemoglobin concentration (g/dl)	32.50 ± 5.94	32.43 ± 2.23	30.58 ± 4.81	0.103
Hematocrit (%)	16.27 ± 5.54	30.08 ± 3.08	44.82 ± 5.24	<0.001
RDW-CV (%)	33.68 ± 5.87	16.30 ± 1.91	13.10 ± 1.09	<0.001
Mentzer index	34.61 ± 17.45	12.33 ± 2.48	20.94 ± 2.29	<0.001

Red cell indices, including mean corpuscular volume (*P* = 0.916), mean corpuscular hemoglobin (*P* = 0.511), and mean corpuscular hemoglobin concentration (*P* = 1.0) did not show significant differences between the ThM group and ThT group. Mean hematocrit was significantly higher in the ThT group than the ThM group (*P* < 0.001), whereas red blood cell distribution width (*P* < 0.001) and the Mentzer index (*P* < 0.001) were significantly higher in the ThM group than in the ThT group (Table 2).

sTfR (*P* < 0.001), ferritin (*P* < 0.001), and visfatin (*P* < 0.001) concentrations were significantly higher in the ThM group than in the ThT group (Table 3).

**Table 3:** Comparison of sTfR, ferritin, and visfatin levels

	ThM group (*n* = 12)	ThT group (*n* = 28)	Control group (*n* = 10)	*P* value
sTfR (ng/ml)	1.181 ± 0.068	0.966 ± 0.093	0.769 ± 0.272	<0.001
Ferritin (ng/ml)	2085.38 ± 723.68	41.40 ± 36.80	61.83 ± 18.81	<0.001
Visfatin (ng/ml)	73.43 ± 11.98	49.33 ± 7.51	36.54 ± 15.04	<0.001

**Figure 1:**
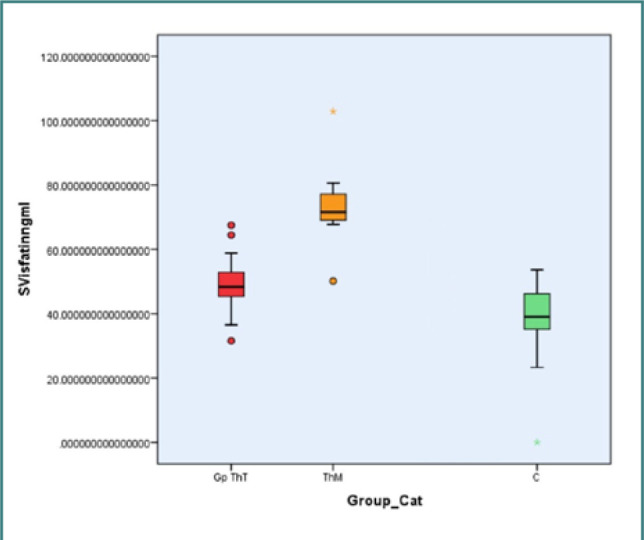
Serum visfatin levels among study participants

Serum visfatin levels were the highest in the ThM group (73.429 ± 11.980 ng/ml), followed by the ThT group (49.336 ± 7.518 ng/ml) and the control group (36.540 ± 15.405 ng/ml), with a statistically significant difference between the different groups (*P* < 0.001). The receiver operating characteristic analysis revealed that serum visfatin levels were significantly different between the three groups (Figure 1).

## DISCUSSION

The absence of the specific globin gene cannot fully explain the variety of conditions associated with thalassemia, such as cardiovascular and chronic inflammatory disorders. The pathogenesis of endothelial damage and vascular inflammation involves several factors, including chronic hemolysis, increasing adherence of erythrocytes and platelets to endothelial cells, oxidative stress, and chronic iron overload [11,12]. Endothelial cells possess a diverse array of roles that enable their involvement in numerous disease processes, such as atherosclerosis, pulmonary hypertension, and chronic inflammation in hemoglobinopathies, including β-thalassemia. Patients with β-thalassemia frequently experience vascular problems that are linked to signs of malfunction and endothelial cell activation [13,14]. The activation of endothelial cells in individuals with β-thalassemia is attributed to increased levels of TNF-α, IL-1β, and vascular endothelial growth factor (VEGF) [15,16]. Furthermore, it has been observed that individuals with β-thalassemia exhibit increased adherence of red blood cells and leukocytes to endothelial cells, which contributes to the development of atherosclerosis and proinflammatory responses. This enhanced adherence is attributed to the production of cytokines and the expression of adhesion molecules by these cells [17–19].

In the present study, the concentrations of sTfR and serum ferritin were found to be significantly higher in the ThM group compared to the ThT group and the control group (*P* < 0.001). A study conducted by Jayaranee *et al*. reported that sTfR levels in individuals with thalassemia were higher, but the difference was not significant [20]. Another study reported that serum ferritin levels were significantly higher in ThM but not in ThT and in the control group (*P* < 0.001) [21].

The expression of visfatin is increased in response to infection, hypoxia, and inflammatory cytokines, and it has the potential to subsequently enhance the activation of the inflammatory cascade [20,21]. The current study provides evidence that individuals diagnosed with β-thalassemia major and β-thalassemia minor exhibit elevated levels of visfatin in their serum compared to healthy individuals. Previous research has also shown that visfatin levels are significantly higher in patients with β-thalassemia minor compared to controls (*P* = 0.031), and in patients with β-thalassemia major compared to those with β-thalassemia intermedia (*P* = 0.002) and β-thalassemia minor (*P* = 0.005) [7].

A study conducted by Abdelwahab *et al*. compared serum visfatin levels between 41 patients with thalassemia and a control group of 21 healthy individuals. They found that patients with β-thalassemia major (*P* < 0.001) and intermedia (*P* < 0.001) had significantly higher concentrations of serum visfatin than the controls [21]. These findings lend even more credence to the concept that an upregulated inflammatory cascade and an elevated level of proinflammatory markers have a significant part in the pathogenesis of the disease.

The study’s limitations include the fact that it was limited to only one region and had a small sample size. Also, the evaluation of endothelial cell function and other adipocytokines was not performed.

## CONCLUSION

The findings of the study show a novel relationship between increasing concentrations of sTfR and visfatin and the severity of β-thalassemia. Visfatin may substantially increase the severity of β-thalassemia by activating pathways associated with inflammation. Mitigating the proinflammatory effects of visfatin may be an intriguing option in the treatment of β-thalassemia. To understand the inflammatory process in β-thalassemia and its effects, it is recommended to study the link between more proinflammatory markers in patients with this genetic condition.
